# Screening PLHIV for depression using PHQs: A RCT comparing non-selective with selective screening strategy within a primary health care facility in Uganda

**DOI:** 10.1371/journal.pone.0270175

**Published:** 2022-06-29

**Authors:** Paul Okimat, Dickens Akena, Denis Opio, Tobius Mutabazi, Emmanuel Sendaula, Fred C. Semitala, Joan N. Kalyango, Charles A. Karamagi

**Affiliations:** 1 Clinical Epidemiology Unit, School of Medicine, College of Health Sciences, Makerere University, Kampala, Uganda; 2 Soroti District Local Government Health Department, Uganda; 3 Institute of Public Health and Management, Clarke International University, Kampala, Uganda; 4 Department of Psychiatry, Makerere University College of Health Sciences, Kampala, Uganda; 5 Department of Internal Medicine, College o Health Sciences, Makerere University, Kampala, Uganda; 6 Makerere University Joint AIDS Program (MJAP), Kampala, Uganda; 7 Department of Pharmacy, College of Health Sciences, Makerere University, Kampala, Uganda; 8 Department of Paediatrics and Child Health, College of Health Sciences, Makerere University, Kampala, Uganda; University of East Anglia, UNITED KINGDOM

## Abstract

**Background:**

Depression is rarely screened for among People Living with Human Immunodeficiency Virus (PLHIV) although it is 2 to 3 times more prevalent among PLHIV than in the general population. In instances where depression is screened for using screening tools, it usually follows noticing depression risk factors. This practice of selectively screening for depression could be leaving some cases of depression unattended to. On the other hand, subjecting every client to screening tools (non-selective screening) offers every patient an opportunity to be managed for depression. However, this could require additional resources as compared to selective screening. We present and discuss results on whether non-selective and selective screening strategies differ in depression case detection, and in addition, we also present perceptions of the stake holders on the two screening strategies.

**Methods:**

The study was conducted in Princess Diana Memorial Health Centre IV HIV clinic using a randomized controlled trial with a qualitative component. To determine whether there was a difference in depression case detection, consecutively sampled participants were randomly allocated to either non-selective or selective screening strategy. Participants allocated to selective screening were screened for depression using the patient health questionnaire (s) (PHQs) if they were at “crisis points”. While those allocated to non-selective screening were screened regardless of whether the “crisis points” were noticed or not. The PHQ-2 and PHQ-9 were used in sequence. 326 PLHIV participated in the study. Outcomes of the MINI evaluation were analyzed for those with PHQ-9 scores of 10 or more to confirm major depressive disorder (MDD). Data was analyzed using the two sample Z-test for proportions with Stata 2013 software. To explore the perceptions of the stake holders, key informant interviews were performed with six stakeholders that experienced the study.

**Results:**

Cases of depression (PHQ-9 score ≥ 5) were more likely to be detected by the non-selective screening strategy 30.2% (49/162) compared to the selective screening strategy 19.5% (32/164) (difference in proportions 0.107, 95% confidence interval 0.014–0.200, Cohen’s h = 0.25, P = 0.03). The stake holders thought it was important to screen for depression among PLHIV with preference to non-selective screening strategy.

**Conclusion:**

Evidence from this data suggests that more cases of depression (PHQ-9 score ≥ 5) are likely to be detected with non-selective screening as opposed to selective screening.

**Trial registration:**

PACTR201802003141213 (name: comparison of routine versus selective screening for depression strategies among PLHIV attending Princess Diana Memorial Health Centre iv Soroti).

## Introduction

Depression is among the leading cause of disability worldwide with over three hundred million people affected [1]. Despite the high burden of depression and negative consequences associated with it, about 46% to 50% of cases of depression are missed in primary care settings in developed countries [2] and close to 100% in developing countries [3–5]. This is due to the fact that depression is not regularly screened for hence leading to missed cases of depression and a lack of data on depression to aid planning [4].

Sub -Saharan Africa is home to over 70% of PLHIV [6] and depression is 2 to 3 times more common among this population than in the general population [7]. The consequences of untreated depression among PLHIV are dare as it can affect adherence to treatment, treatment response [8]; correct consistent condom use [9]; ability to work, retention into care, and the general wellbeing of the depressed PLHIV [10, 11]. However, depression treatment is rarely integrated into HIV care, partly due to the scarcity of mental health professionals, poor mental health literacy, lack of knowledge on effective mental health provision models, among others [12, 13].

As governments in sub-Saharan Africa attempt to integrate screening for depression among PLHIV, there is limited information on the most appropriate strategies on how to integrate this service into care and more so the benefits that come along with each strategy [11]. Some studies have focused on comparing the case detection, and treatment out comes especially between routine (non-selective) and regular clinical practice (“clinical acumen”) [14] while one considered comparing selective screening (systematic screening or targeted screening) and clinical acumen [15]. However, we are not aware of any study comparing non-selective screening and selective screening strategies.

The primary objective was to determine whether there was a difference in cases of any depression (PHQ-9 score ≥ 5) across the study arms. The secondary objective was to determine whether there was a difference in detection of cases of major depression across the study arms. We explored for the presence of trend by depression grade across study arms. In addition, we sought to describe the perceptions of the stakeholders on the two screening strategies.

We had hypothesised that screening all PLHIV aged 18 years and above for depression on every clinic visit using the PHQ-2 and PHQ-9 (in sequence) reduces the missed cases of depression by at least 10% during the screening period.

Selectively screening for depression among PLHIV could be a viable option for resource constrained settings as it could perhaps offer similar case detection, reduce false positives, and lessen time required to screen the entire clinic for depression. On the other hand, non-selective screening [16] could minimise the cases of depression missed since it offers an advantage of most PLHIV being screened for depression if well implemented. It may however come with a demand for more resources. We believed that the study would offer information necessary in developing the policy in relation to screening for depression among PLHIV.

## Materials and methods

### Study design and setting

We conducted a randomized controlled trial with a qualitative component. To determine if there was a difference in depression case detection, participants were randomly allocated to either non-selective or selective screening strategy. The qualitative study was done to determine the perceptions of stakeholders on screening for depression to explore the results from the quantitative study and identify the potential barriers and facilitators to screening for depression.

The study was conducted in Princess Diana Memorial Health Centre IV a government health facility found in Soroti district in the north-east of Uganda. The prevalence of HIV in north-east Uganda is 3.7% [17]. Princess Diana Memorial Health Centre IV is in a peri-urban setting within northern division along Soroti- Moroto road. Northern division had a population of 19,382 people [18]. Princess Diana Memorial Health Centre IV had a catchment population of approximately 11,500 people and approximately 470 HIV active positive patients. One study reported the prevalence of depressive symptoms in eastern Uganda among people living with HIV to be 46.8% [19]. The Health Centre was staffed with two psychiatric nurses serving the mental clinics in addition to the other non-psychiatric staff. The research assistants were selected from the health facility trained for one day, and on job guidance for three days.

### Quantitative data collection

Participants were randomly allocated to either non-selective or selective screening strategies using a one to one allocation ratio. The quantitative data was used to determine whether there was a difference in case detection across the study groups. Screening was done in two separate consultation rooms with each room assigned to a screening strategy.

### Participants

The study population was PLHIV attending the HIV clinic of Princess Diana Memorial Health Centre IV in Soroti district Uganda. Only adults 18 years and above who consented to participate were included. All PLHIV who were too ill to withstand study procedures were excluded from the study.

### Interventions

#### Non-selective screening strategy

Note: in this manuscript, we use the name **‘non-selective screening’** as opposed to **‘routine screening’** in the protocol, and registry. This is because the former name was found to describe the strategy better.

**Fig 1:**
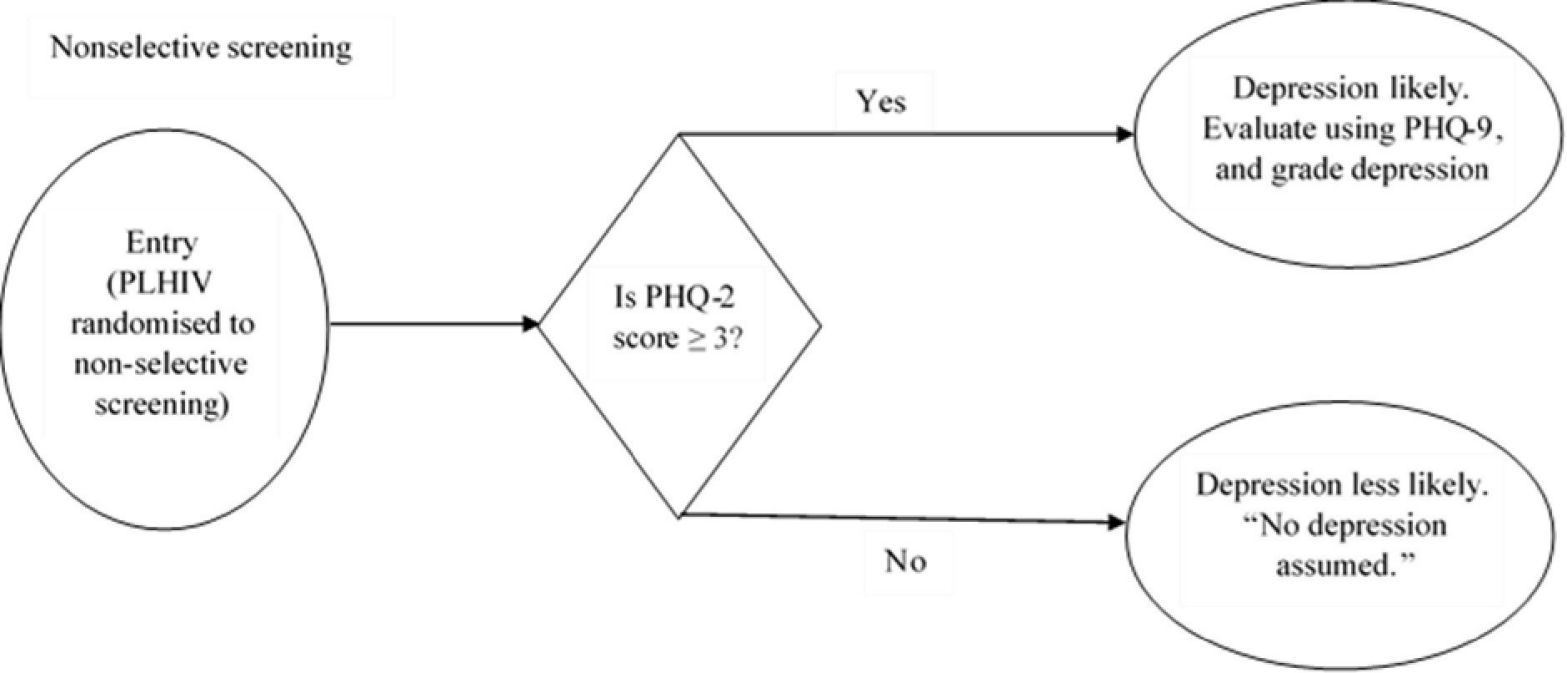
An illustration of non-selective screening strategy among PLHIV.

All PLHIV who were randomly allocated to this strategy were subjected to the PHQ-2 and if the patient scored a value greater than or equal to 3, he / she was then subjected to PHQ-9 (Fig 1). The screening was done in a room dedicated to non-selective screening strategy by a clinician assigned to the strategy.

After, all participants were evaluated using the Mini international neuropsychiatric interview (MINI) in a room dedicated to the MINI. The results of the MINI were used to confirm major depressive disorder (MDD) in those patients with PHQ-9 scores of 10 and above.

#### Selective screening strategy

**Fig 2:**
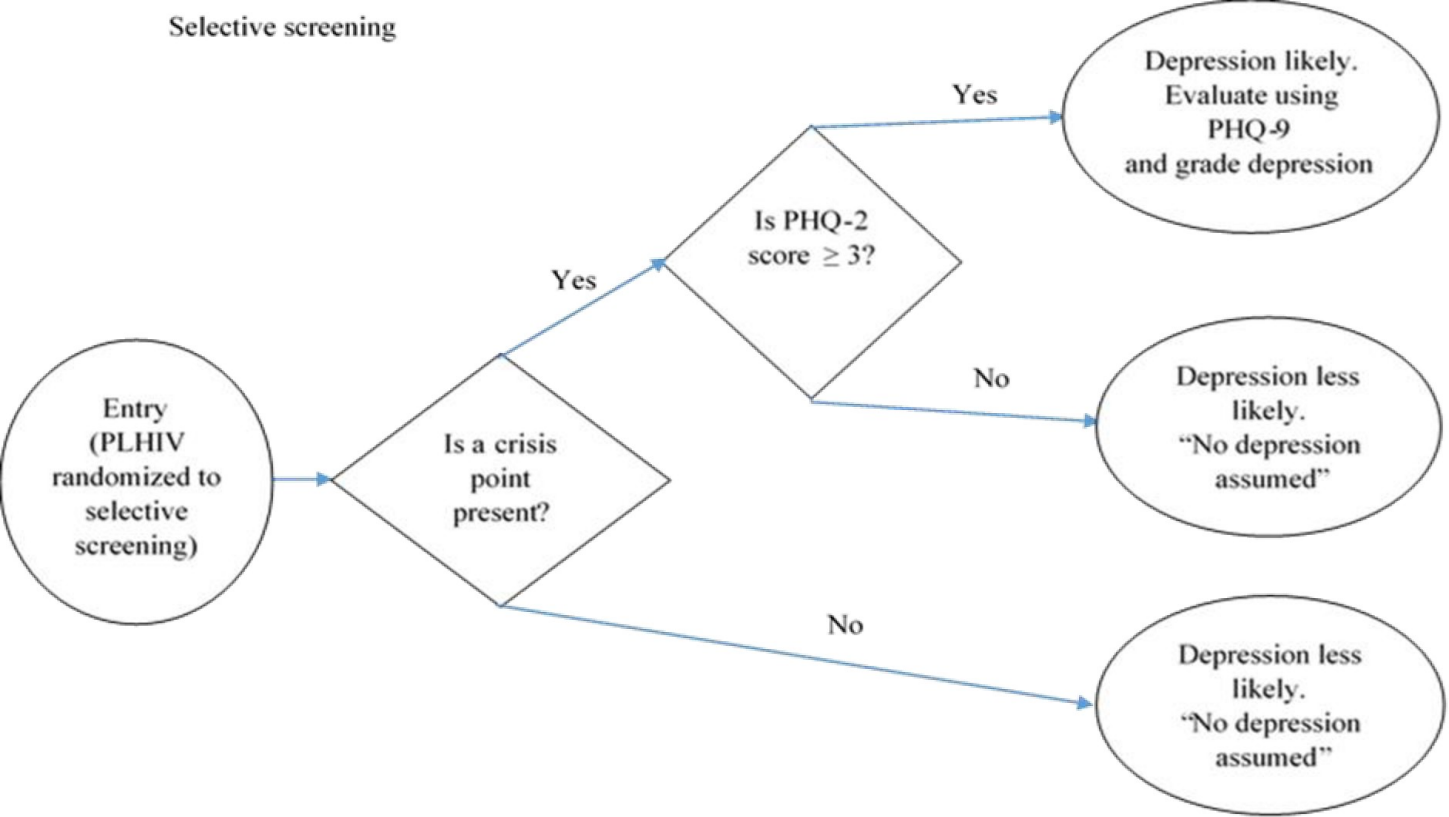
An illustration of selective screening strategy for depression among PLHIV.

The control arm provided the “selective screening” strategy. A participant randomly allocated to selective screening was subjected to the PHQ-2 and later (if PHQ2 ≥3) PHQ-9 when the patient was at the “crisis points of life” (Fig 2). The following were considered to be crisis points: newly diagnosed with HIV or at disclosure of HIV status, occurrence of any physical illness, chronic symptoms, progression of disease or hospitalisation or diagnosis of AIDs, introduction to antiretroviral therapy, death of a significant other, necessity of making end of life, and permanency planning decision, major life changes like child birth, pregnancy, loss of a job, and end of a relationship [20]. In the implementation of the study, ‘chronic symptoms’ instead of any ‘new symptoms’ (as stated in the protocol) were considered. This was based on evidence from literature that suggested that chronic symptoms were associated with depression [21].

The clinician was to commence to subject the PHQ-2 to the client / patient upon noticing a “crisis point”. The health workers were trained to assess for crisis points. These crisis points were obtained from the Uganda HIV prevention and treatment guidelines 2016. The screening was done in a room dedicated to selective screening strategy by a clinician assigned to the strategy. After, all participants were evaluated using the Mini international neuropsychiatric interview (MINI) in a room dedicated to the MINI. The results of the MINI were used to confirm major depressive disorder (MDD) in those patients with PHQ-9 scores of ≥10 and to capture any otherwise missed MDD cases for management.

### Outcomes

The outcome variable was cases of depression detected.

#### Case identification

The PHQ-2 tool is a two-item instrument that identifies the frequency of depressed mood and anhedonia over the past two weeks. The purpose of the tool is to screen for depression in a first step approach. The PHQ-2 score ranges between 0–6. Those with a score equal or greater than 3 are further evaluated using the PHQ-9 [22]. The PHQ-9 is a 9-item depression screening instrument that determines the presence and frequency of the 9 core depressive symptoms identified in the DSM-IV over the previous 2 weeks. Scores range from 0–27. Categories of severity of depression are arrived at following the diagnosis PHQ-9 guide [23]. One was considered depressed if one had a PHQ-9 score of 5 and above. Following the PHQ-9 guide, scores of 0–4, 5–9, 10–14, 15–19, and 20–27 represented cut-off ranges for none, mild, moderate, moderately severe, and severe depression, respectively. This tool has been tested in sub-Saharan Africa and found to be suitable for use among PLHIV [24]. The depression cases were graded based on the PHQ guide. In addition, a person with a PHQ-9 score of 10 and above was confirmed for MDD using the MINI results.

### Other variables

The variables age, marital status, sex, weight, viral load of the participants, alcohol consumption, and cigarettes smoking status were collected as baseline characteristics. Alcohol consumption and cigarettes smoking status had not been prespecified in the protocol but were later on taken up after considering them as potential confounders.

### Treatment of the participants

All participants found to be suffering from depression were treated basing on the Uganda national HIV prevention and treatment guidelines. Participants either received counselling or antidepressants. These participants were followed up in their subsequent clinic visits to monitor the prognosis. However, this paper does not present or discuss the results of the treatment outcome given that the study focused on case detection, and health care perceptions on the screening strategies.

### Sample size estimation and sampling

A sample size of 288 PLHIV was arrived at using a Z-test sample size formula for two independent proportions by Beth Dawson, and Robert G. Trapp [25]. The power and level of significance considered were 90% and 5% respectively. It was assumed that non-selective screening would realise a reduction in cases of depression missed by at least 10% [26] from approximately 95% of the cases of depression which are assumed to be missed in Africa [4]. 20% adjustment was made to cater for non-response increasing the sample for 350 PLHIV. The participants were sampled and enrolled consecutively as they met the eligibility criteria.

### Randomisation

We were able to randomly assign 326 (out of 350) PLHIV to either selective or non-selective screening strategies in randomly varying block sizes of 4, 6, 8, 10, and 12. The randomisation code was concealed using the sequentially numbered opaque sealed envelopes. This was generated by an independent statistician and administered by the HIV clinic nurse In-charge who enrolled the patients consecutively as they met the eligibility criteria.

### Blinding

The study was single blinded. Participants were blinded while the clinicians administering the strategies were not blinded (it was not feasible to blind them). Patients were not told which study arm they had been allocated to.

### Statistical analysis

Data was analysed using Stata 2015 using intention to treat analysis. The skewed continuous data was summarised using median and quartiles while non-skewed data was summarised using mean and standard deviation. The categorical variables were summarized using frequencies and proportions. The Z-test for two independent proportions was used to compare the proportion of depression case detection between the groups. We also explored for a trend in depression grades/severity using Cuzick’s non-parametric test for trends.

### Qualitative data collection

To describe the perceptions of stakeholders on the screening strategies for depression among PLHIV, voice recorded key informant interviews were conducted among six purposively sampled health workers. The 6 health care workers who were requested to participate in the qualitative study had participated in the screening process. Perceptions were opinions, thoughts, views, beliefs, or feelings of stake holders about the screening for depression, and the strategies employed. The Theoretical Domains Framework (TDF) was used in generating the guide and formulating questions to explore the perceptions of the health workers on screening for depression. In addition, the responses of the participants were latter mapped against the domains in the framework.

### Qualitative analysis

Voice recorded interviews were transcribed verbatim and coded by two independent research assistants manually. During the coding process, discrepancies or disagreements were discussed until consensus was reached. In cases where the consensus was not reached on which domain to allocate the text, the text was allocated to the two domains. The information from the qualitative analysis was triangulated with the quantitative data during the discussion of results.

### Ethics

Ethical approval to conduct this study was sought from the Makerere University School of Medicine Research and Ethics Committee approval number is REC REF 2018–041. The study was registered with the Pan African Clinical trial registry with a registration number of PACTR201802003141213. The protocol may therefore be obtained from the same registry. We also obtained administrative permission from Soroti Municipal Council, and the in-charge of Princess Diana Memorial Health Centre IV. All study participants provided informed consent (written or witnessed thumb print). Access to data was restricted to only the study team.

## Results

### Participant flow

**Fig 3:**
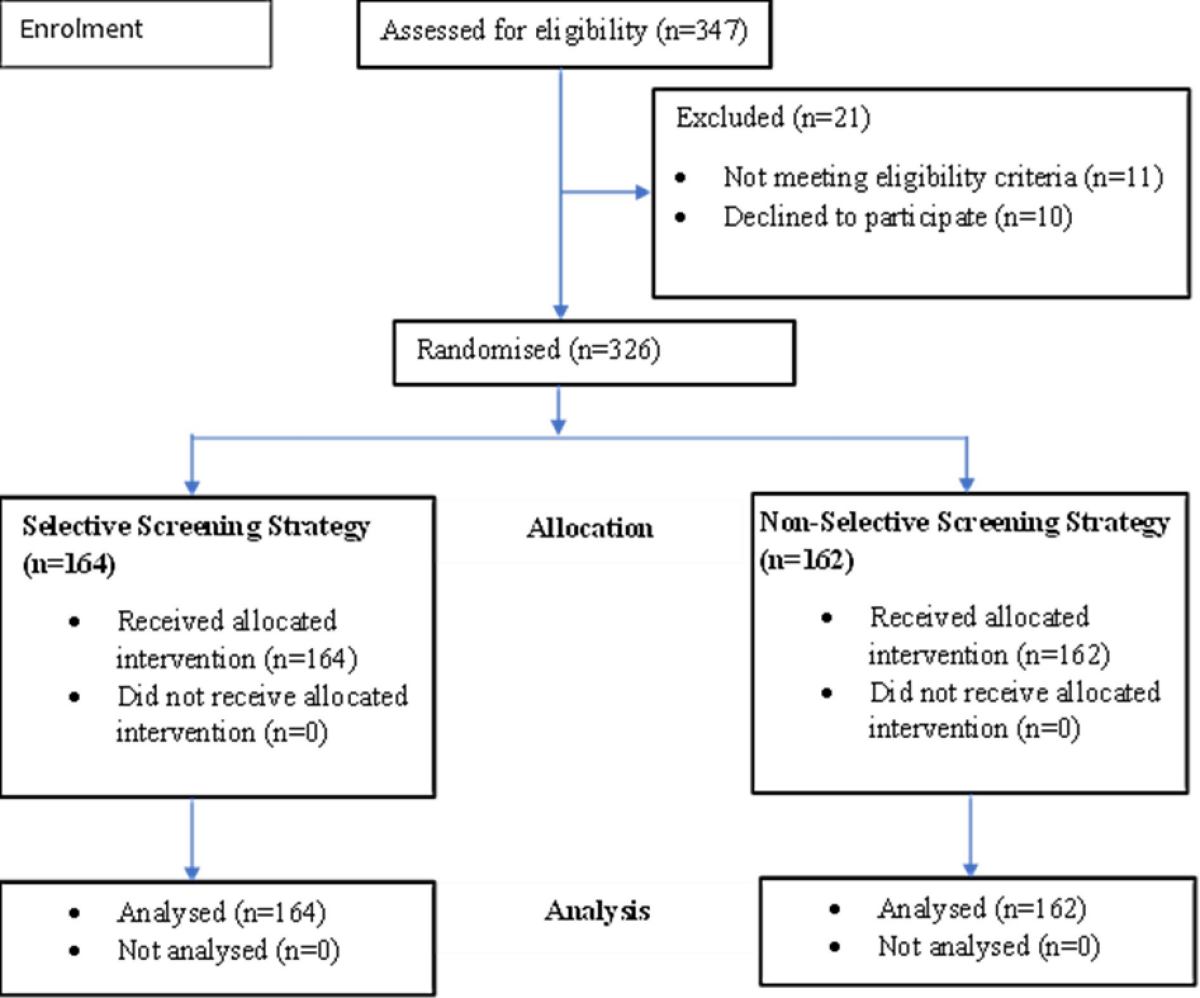
Study profile of PLHIV at Princess Diana Memorial Health Centre IV, Soroti District, who participated in the study from April to June 2018.

Out of 347 participants that were assessed for eligibility, 326 were randomised and analysed (Fig 3).

This study was carried out from April 2018 to June 2018. The data entry was completed in September 2018 after exceeding the required 288. No harm arising from participating in the study was reported.

### Description of the study population that participated in the quantitative study

Overall, the baseline characteristics were distributed evenly with no significant difference across groups for all potential confounders as shown in the Table 1.

**Table 1:** Baseline socio-demographic and clinical characteristics of 326 participants at Princess Diana Memorial Health Centre IV, Soroti District, April to June 2018.

Variable		Selective screening	Non-selective screening
**Number of participants**		164	162
**Age**	Median	35.5	37
	IQR (Q_1,_ Q_3_)	10(30,40)	15(30,45)
**Weight**	Median	54	56
	IQR (Q_1,_ Q_3_)	11(50,61)	10(50,60)
**Sex**	Females. Number (%)	97(59.1%)	90(55.6%)
**Marital status**	Number (%);		
	• Not married	49(29.9%)	49(30.2%)
	• Married	115(70.1%)	113(69.8%)
**Alcohol consumption**	Alcohol consumers. Number (%)	57(34.8%)	42(25.9%)
**Smoking cigarettes**	Smoke cigarettes	12(7.3%)	12(7.4)
Viral load*	Participants with non-suppressed viral load (VL>1000copies/ul). Number (%)	17(11.7%)	13(9.0%)

*Missing values- 19 in selective screening group, and 17 in the non-selective screening group.

Only one case of severe depression was identified, and this was by non-selective screening strategy. The proportion of MDD correctly identified by non-selective screening arm was 22% (35/162) while that by selective screening was 14% (23/164). The proportion difference between the groups was 0.08 (Cohen’s h = 0.20), p-value 0.06 (two-sided) and 95% confidence interval of (-0.163,0.003).

For any depression (PHQ-9 score ≥ 5), it can be noticed that more cases of depression were detected by non-selective screening strategy as compared to selective screening strategy 30.2% (49/162) versus 19.5% (32/164) respectively. Overall, the difference in proportion was 0.107 (Cohen’s h = 0.25) with a p-value of 0.03, and 95% confidence interval of (-0.200, -0.014). Grade of depression was not significantly different between groups (Table 2).

**Table 2:** Grading of cases of depression detected by non-selective or selective screening as measured by the PHQ9 among 326 participants at Princess Diana Memorial Health Centre IV, Soroti District, April to June 2018.

Grade of depression	Selective screening Number (%)	Non-selective screening Number (%)	Total	P-value
**No depression**	132 (80.5)	113 (69.8)	245 (75.2)	0.07
**Mild depression**	5 (3.0)	8 (4.9)	13 (4.0)	
**Moderate depression**	15 (9.1)	29 (17.9)	44 (13.5)	
**≥ Moderately Severe depression**	12 (7.3)	12 (7.4)	24 (7.4)	
**Total**	164 (100)	162 (100)	326 (100)	

Cuzick’s non-parametric test for trends was used.

### Description of the study population that participated in the qualitative study

Six (6) HIV health workers selected from within the Health Centre participated in the study. These included 2 general practitioners, 2 psychiatric nurses, 1 village health team member, and 1 expert client. Of these, 2 were male and 4 were female. Participants ranged from 30 to 40 years of age with a mean age of 35 years. All interviews were conducted in the English language. The respondents had at least three years’ work experience as HIV health care workers. A total of 6 key informant interviews were conducted over a week’s period.

### The perceptions of health workers on screening for depression and the screening strategies

#### Skills

Some health workers believed that screening for depression was easy.

*“……both selective and non-selective screening strategies are easy*, *except one seems to be more detailed than the other*, *…*.*”*, a key informant health worker from PDMHCIV 2018.

#### Social / professional role and identity

All the health workers believed that screening for depression was a part of their profession and could therefore be a part of the work though there was a differing view that the role was more inclined to the field of psychiatry.

*“……*. *screening for depression is every one’s role in this health facility I just think people need to be reminded of their roles…*.*”*, a key informant VHT from PDMHCIV 2018

#### Beliefs about capabilities

Some participants felt they were competent enough to screen treat and manage depression.

*“…*‥ *I believe I am able to screen for depression; I can also teach other people how to screen for depression if someone needs to be trained……”*, a key informant health worker from PDMHCIV 2018.

#### Beliefs about consequences

Though health workers believed the screening for depression would benefit PLHIV there was a concern that selective screening would live out some cases of depression. There was also a concern of patients not cooperating during the screening process.

*“…sometimes patients pretend*, *you ask them questions and they tell you a different thing and yet on the other side you see all the features*. …*”*, a key informant health worker from PDMHCIV 2018.

*“…for selective screening I believe there is a possibility that some patients will be left out because you select only a few…”*, a key informant health worker from PDMHCIV 2018.

#### Environmental context and resources

The health workers felt that in as much as screening for depression is possible, some situations or resources could affect the screening process.

*“…you know the problem is that this health facility is small*, *and some patients don’t want to be seen so they come with an intention of getting refills quickly……*.*”*, a key informant patient 2018.

*“…if each patient is to receive a questionnaire*, *then primary health care funds may not be able to sustain the process…*.*”*, a key informant health worker from PDMHCIV 2018.

#### Behavioral regulation

Health workers suggested that the health information management system be improved to capture the depression in this case by including a column for depression assessment in the HIV care cards, capturing the disease in the HIV register.

*“…this condition should be tracked in the HIV care card and registers…it will be mandatory to screen…*.*”*, a key informant health worker from PDMHCIV 2018.

Domains of optimism, emotions, intentions were not found to be relevant in the study. These domains were not field with texts that differed from the other domains, so the researcher opted to leave them out.

## Discussion

By randomising the study participants to study arms, we intended to distribute all study characteristics (including the categories of depression) uniformly to either study arms. Non-selective screening detected more cases of depression than selective screening strategy. The differences between groups in MDD cases detected, and severity/grade, were not statistically significant at the 5% level.

The difference in the cases detected between groups could have risen due the difference in screening steps applied. In the non-selective screening strategy two steps were used, while in the selective screening strategy, three. Since sensitivity reduces when tests are applied in series [27], the extra step in selective screening strategy is thought to have contributed towards the reduction in sensitivity hence the difference in cases of depression detected.

It is also worth appreciating that selective screening detected cases of such magnitude. This could have arisen due to a number of factors, first and foremost the Hawthorne effect [28] and secondly the motivation provided to the research assistants. The motivation in form of the regular supervision [29], and the remuneration [30] could have prompted vigilance during the screening period. Given that the crisis points covered many risk factors and triggers of depression, we would expect literally no difference in cases of depression detected across study groups. However, it is possible that the findings of the study could have been affected by the fact that the attending health workers were multitasking / had competing responsibilities at the point of execution of the study [31]. This practice of multitasking in addition to the other competing responsibilities (which is common in public health facilities) could have affected the outcome of results [32]. Therefore, under a well monitored conducive environment, and appropriate motivation, it is possible that selective screening can perform better.

In addition, other factors such as cigarette smoking, alcohol consumption, and culture could affect depression screening outcomes. Given that alcohol consumers present at times with defensive characteristics such as denial, minimisation, projection, etc, [33] health workers if not keen, could miss diagnosing depression. On the other hand, cigarette smokers smoke to reduce or escape unpleasant feelings (feelings of depression) [34] which perhaps could lead into misleading screening outcomes. The other possible reason is that culture influences the way patients express themselves to clinicians. For example, it is well known that men are less likely to express pain or emotional symptoms / grievances except when severe.

Some studies have been done to compare routine (“non-selective”) and clinical acumen, these studies realised a statistical difference indicating that routine screening realised more cases of depression as compared to clinical practice [14, 35]. However, there is hardly any study (that we are aware of) comparing non-selective and selective screening approaches. The only study that we are aware of that had tested a screening criterion (Systematic screening) similar to the one referred to as selective screening in this study was done is Spain [15]. No difference was realised between groups and one of the possible reasons noted for this lack of difference was the failure to adhere to the study protocols.

By large the thoughts of health workers were positive towards screening for depression with preference towards non-selective screening as compared to selective screening. However, one health worker believed that selective screening could be offering a more comprehensive consultation time (/ interaction with the patient) compared to non-selective screening given that it requires that one explores the social, physical, cultural and economic life of the patients during the process of selecting the patients with / at crisis points. Some concerns however arose regarding knowledge, beliefs about consequences, and environmental context and resources.

Regarding the beliefs about consequences, health workers raised concerns about the possibilities of patients of depression being missed out by selective screening. This is supported by the overall cases of depression detected by the different strategies as shown in the results section. As suggested, it is possible that providing for sections to monitor depression in the HIV care cards could further improve depression detection as this could compel screening for depression in every visit.

This perception towards screening for depression is not unique to this setting as other studies have found similar findings among which are the studies done in Uganda among HIV counsellors [36], and in India among health workers [37]. The findings of the study are however different from some findings of a systematic review of studies done among primary care physicians in the United States of America where some of the attitudes / thoughts towards screening for depression were found to be negative [38].

As pertains the environmental context and resources, there was a concern of the effects of stigmatization. Patients (PLHIV) demand to be served quickly to minimize the chance of being noticed by other people. This could affect screening time and in turn affect the outcomes of screening for depression [39, 40]. There was also a concern that stationery could be affected by PHC funds given that the funds are neither adequate nor regular [41, 42] therefore leading to a shortage in screening materials. In view of the above qualitative findings, screening for depression should be accompanied with improvement in the health information management system (to capture and track cases of depression among PLHIV), training of health workers on screening for depression, and mass sensitization of the public on depression the dangers that come along with it.

This study is not without limitations. In as much as training of the staff was done prior to study commencement, and a reference list of crisis points provided, it was not feasible to assess whether the health professionals were strictly following the screening process allocated to them while attending to the patients. Since the study was conducted among adults aged 18 years and above, these findings may not be generalizable to those bellow 18 years of age. There was also concern about the adequacy of the sample size to detect differences between grades of depression. We also think the number of key informants interviewed was small and perhaps a bigger number could have yielded more varying views.

Nevertheless, this data suggests that selectively screening for depression among PLHIV basing on risk factors can lead to missing some patients that could have benefited from depression care. In addition, the high response from the participants and willingness to participate in the study minimized the selection bias hence increasing generalizability among this age group in similar settings. Findings of this study should be interpreted with consideration of findings from other studies. A pragmatic trial with a larger sample size could help shine more light on the results and rule out random error.

## Conclusion

Evidence from this data suggests that PLHIV could benefit more from non-selective screening as opposed to selective screening strategy. Implementing screening for depression (regardless of the strategy used) should be accompanied with improvement in the health information management system (to capture and track cases of depression among PLHIV), training of health workers on screening for depression, and mass sensitization of the public on depression and the dangers that come along with it.

## Supporting information

S1 Checklist CONSORT 2010 checklist of information to include when reporting a randomised trial*. (DOC)

S1 Protocol (PDF)
