# Supplementary material for: Screening PLHIV for depression using PHQs: A RCT comparing non-selective with selective screening strategy within a primary health care facility in Uganda
Source: PLoS One. 2022 Jun 29;17(6):e0270175. doi: 10.1371/journal.pone.0270175 (PMC9242435; doi:10.1371/journal.pone.0270175)
Supplement: S1 Protocol — (PDF) [file pone.0270175.s002.pdf]

Study Protocol

Comparison of Routine Versus Selective Screening for Depression Strategy Among PLHIV Attending Princess Diana Memorial Health Centre IV Soroti.

Paul Okimat

Supervisors
Dr. Dickens Akena
Assoc. Prof. Charles Karamagi.

Date of approval by IRB: 15th / March / 2018

LIST OF ACRONYMS

AIDS	Acquired Immune Deficiency Syndrome
ART	Antiretroviral Therapy
CES-D	Centre for Epidemiologic Studies Depression Scale
CD4	Cluster of Differentiation 4
HAART	Highly Active Antiretroviral Therapy
HSCL	Hopkins Symptom Checklist
HIV	Human Immunodeficiency Syndrome
MDD	Major Depressive Disorder
MINI	Mini International Neuropsychiatric Interview
MOH	Ministry of Health
PHQ-2	Patient Health Questionnaire-2
PHQ-9	Patient Health Questionnaire-9
PLHIV	People Living with Human Immunodeficiency Syndrome
WHO	World Health Organisation

OPERATIONAL DEFINITIONS

Depression Based on the PHQ-9 scores depression will be categorised as; scores of (0-4)-none, (5-9)-mild, (10-14)-moderate, (15-19)-moderately severe, and (20-27)-severe depression (Spitzer R, et al).

Based on the MINI; MDD, current, 5 or more answers (**A1-A3**) coded **yes.** MDD, recurrent (**A4)** coded yes (following diagnosis of major depressive disorder current) (Sheehan DV & Lecrubier Y, 1992).

Screening The presumptive identification of unrecognized cases of depression by the appropriate application of the PHQ2 and PHQ9, examinations, or the M.I.N.I.

Routine screening Screening every PLHIV attending the HIV clinics at every visit using the PHQ-2 and PHQ-9.

Selective screening This is targeted screening of PLHIV at high risk offered when a health provider finds it appropriate to screen especially when at crisis points of life.

Crisis points of life These include: newly diagnosed with HIV or at disclosure of HIV status; occurrence of any physical illness, recognition of new symptoms / progression of disease or hospitalisation or diagnosis of AIDs; introduction to medication; death of a significant other; necessity of making end of life; and permanency planning decision, major life changes like child birth, pregnancy, loss of a job, and end of a relationship (MOH Uganda, 2016).

ABSTRACT

Introduction: People living with HIV are at risk of developing depression. Depression has been found to worsen their wellbeing, the ability to adhere to treatment, to increase loss to follow up among others.

The approach recommended by the ministry of health of "selective screening" could be living a gap in detection in screening for depression given that it does not cover all the risk factors. The alternative routine screening offers an advantage of all PLHIV to be screened on every clinic visit though with more resources required. Knowing how to strategize screening for depression will contribute towards the knowledge needed to develop guidelines for screening for depression among people living with HIV.

Objective: To determine whether routine screening strategy differs from selective screening strategy in depression case detection, and to describe the perceptions of stakeholders on the strategies for screening depression among PLHIV.

Methods: A mixed methods study design with quantitative and qualitative data collection methods will be used. Quantitative data to determine whether there is a difference in cases of depression detected when using routine or selective screening strategies will be collected from 382 consecutively sampled PLHIV attending Princess Diana Memoria Health Centre IV HIV clinic between January and March 2016.

Qualitative data on perceptions of the health workers on the screening strategies will be captured using key informants' interviews from approximately 5 health workers providing HIV services in the clinic.

Triangulation of qualitative and quantitative literature will be done in the analysis.

Utility: Study findings will create a useful guide for improving current policy and practice on screening for depression among people living with HIV.

CONTENTS

LIST OF ACRONYMS i

OPERATIONAL DEFINITIONS ii

ABSTRACT iii

CHAPTER ONE 7

1.00 Introduction 7

1.01 Problem statement 9

1.02 Justification of the study 10

1.03 Research questions 11

1.04 Objectives of the study 11

General objective 11

Specific objectives 11

1.05 Hypothesis 11

1.06 Theories to be used 12

1.07 Conceptual framework of screening for depression strategy 14

Scope of the study 14

CHAPTER TWO 15

2.00 Literature review 15

2.01 Depression 15

2.02 Screening for depression 16

2.03 Approaches to screening 17

2.04 Studies done on screening for depression strategies / approaches 18

2.05 Perceptions of depression screening 20

CHAPTER THREE 21

3.00 METHODS 21

3.01 Study design..21

3.02 The study setting. 21

Methods for objective one 22

3.03 Population 22

3.04 Eligibility criteria 22

3.05 Sample size estimate 22

3.06 Sampling procedure 23

3.07 Randomisation 23

3.08 Study variables 24

3.09 Data collection 25

3.10 Data management 26

3.11 Data analysis 27

Methods for objective two 27

3.12 Participants 28

3.13 Sampling procedure 28

3.14 Study variables 28

3.15 Data collection 28

3.16 Data management 28

3.17 Data analysis 29

3.18 Quality control 29

3.19 Ethical Considerations 30

3.20 Limitations of the study 30

3.21 Dissemination plans 30

References 32

CHAPTER ONE

1.00 Introduction

Depression is the leading cause of disability worldwide with over 300 million people affected (4.4% of the world's population) (UN health agency, 2017). It is one of the most prevalent mental health comorbidities among People Living with Human Immunodeficiency Virus (PLHIV) (Benton, 2008; Okeke & Wagner, 2013), and it is 2 to 3 times more prevalent among PLHIV in Sub-Saharan Africa (SSA) (Charlotte, Dabis, & de Rekeneire, 2017). In 2016, there were 36.7 million PLHIV worldwide accounting for 0.8% of the world's population aged 15-49 years("Fact sheet - AIDS epidemic status," 2016).

Major depression among PLHIV in the SSA region is estimated to range from 9% to 32% (Charlotte et al., 2017). This burden is in a population with approximately 25.5 million PLHIV ("Fact sheet - AIDS epidemic status," 2016) amounting to 4.2% of the population of the region aged 15-49 year("WHO | (GHO) data," 2017).

Lack of screening and subsequent treatment for mental health disorders can affect general health, quality of life, adherence to antiretroviral therapy (ART) and retention into care (9) and therefore affecting most stages in the HIV cascade of care. Depression has been associated with treatment failure and the emanation of drug resistant HIV strains (Hartzell, Janke, & Weintrob, 2008) and it is partly attributed to poor adherence to ART (Gonzalez, Batchelder, Psaros, & Safren, 2011). Screening for, and treating depression has been found to improve health outcomes(Gaynes et al., 2015).

About 46% to 50% of cases of depression are missed in primary care settings in developed countries(Caballero et al., 2008) and close to 100% in developing countries (Abebaw, 2017; Jenkins et al., 2013; Udedi, 2014).

In Uganda, the prevalence of HIV stands at 6.2% (WHO, 2017b) but varies in different regions of the country from 8% to 46% (Akena, 2013; Nakimuli, 2011; Nakku, Kinyanda, & Hoskins, 2013).

Though the ability of the MOH recommended strategy of “selective screening” to detect depression cases is not known., selective screening for depression could offer an advantage of less time spent on screening for depression and less work load. However, selective screening for depression may leave other PLHIV with depression not diagnosed given that the criteria does not cover all the risk factors.

A study done in a developed country found no difference in the cases of depression detected between selectively screening people at high risk and usual clinical practice (basing on signs and symptoms). Failure to adhere to the screening criteria by the primary care physicians was the explanation for the no difference seen (Romera et al., 2013).

Routine screening for depression among people with HIV was found to be beneficial in not only detecting depression but improving the clinical outcomes as well (Schumacher et al., 2013). Though routine screening offers an advantage of seeing to it that all the PLHIV are screened for depression, it may come with a cost of more time and more work load as compared to selective screening.

Majority of studies done have focussed on effects of notification of patients’ depression status to health workers. A meta-analysis on the effects of notification of patients’ depression status showed an improvement ranging from 10% to 20% was noticed. However, there is hardly any study comparing the two screening strategies of routine and selective screening strategies.

The purpose of this study therefore is to determine whether the selective screening strategy differs from the routine screening strategy in case detections; and to describe the perceptions of the health workers and patients in the study site on the different screening strategies.

1.01 Problem statement

The prevalence of depression among PLHIV in Uganda varies from 8% to 46% (Akena, 2013; Nakimuli, 2011; Nakku et al., 2013).

In spite of this high burden, depression is not routinely screened for and thus likely to be missed. The failure of health workers to detect the cases of depression is as a result of a number of reasons which include; poor mental health literacy, health worker un awareness, high patient numbers, and shortage of health workers (Chibanda et al., 2016). This leads health workers to focus on what is considered urgent or important.

In addition, signs and symptoms of depression mask or mimic the clinical presentations of other physical illnesses thus leading to diagnosis of the condition (Goodwin, 2006). Depression is also at times mistaken to be a reaction to medical illness. However, though understanding depression is important in diagnosing depression, it is important to note that training alone has not been found to be enough to increase case detection (Reynolds & Patel, 2017).

It is also worth noting that in addition to the primary care workers not understanding the manifestations of depression as a mental illness, the patients too do not understand it and may at times mistake it for demonic possession or witchcraft (Korstanje, 2016; Sanganyado, 2017). As a result, the patients may not take initiative to clearly describe or report it as such during clinic visits.

Depression affects the general wellbeing of PLHIV, it is associated with a low CD4 count, poor adherence, and poor retention in care among others therefore affecting every stage in the cascade of care (WHO, 2017).

Routine screening could potentially be beneficial but there is limited information in this setting to support this, given the additional resources required.

1.02 Justification of the study

The MOH through the 2016 consolidated guidelines for prevention and treatment of HIV in Uganda recommends annual screening for depression, screening at presentation of clinical features and at crisis points in life (for example when newly diagnosed with HIV, death of a loved one, etc) among PLHIV. However, this approach targeting those at high risk could be leaving a gap in the ability to detect depression cases among the PLHIV, given that there are other relevant and common risk factors among PLHIV in primary care settings that are left out from the guide for example; age, poverty, social isolation, etc, and in addition the patients are constantly exposed to some risk factors such as treatment with stavudine, efavirenz, and HIV infection itself that are associated with the disease.

The current practice is opportunistic in that patients are screened when a practitioner notices a need or upon request which in most cases is left not followed up.

Routine screening for depression on every visit gives an advantage of screening most if not all PLHIV in case it is appropriately implemented given that even if physical signs or leading symptoms are not reported, screening would still be done. However, selective screening for depression could be offering an equal detection of cases of depression with an advantage of lower work load and time compared to routine screening.

Information from this study will contribute towards the knowledge necessary in the development of policy and guidelines for screening of depression among PLHIV.

1.03 Research questions

a) Is there a difference in cases of depression detected when using routine or selective screening strategies?

b) What are the stakeholders' perceptions on the screening strategies for depression among PLHIV?

1.04 Objectives of the study

General objective

To determine whether routine screening strategy differs from selective screening strategy in depression case detection, and to describe the perceptions of stakeholders on the strategies for screening depression among PLHIV.

Specific objectives

a) To determine whether there is a difference in cases of depression detected when using routine or selective screening strategies among PLHIV.

b) To describe the perceptions of stakeholders on the screening strategies for depression among PLHIV.

1.05 Hypothesis

Screening all PLHIV aged 18 years and above for depression on every clinic visit using the PHQ-2 and PHQ-9 will reduce the missed cases of depression by at least 7.5% during the screening period.

1.06 Theories to be used

The theoretical domains framework

The theoretical domains framework (TDF) is a synthesis of 33 theories of behaviour and behaviour change combined into 14 (12 originally) domains. It is a theoretical framework and not a theory as it is sometimes mistaken to be; it provides a theoretical lens through which to view the environmental, social, cognitive, and affective, influences on behaviour. The frame work does not propose testable relationships between elements. The TDF was originally developed for implementation science research to bring out influences on health professional behaviour related to implementation of evidence-based recommendations(Curran et al., 2013). The theory has a number of domains such as knowledge; skills; social or professional role and identity; beliefs about consequences; beliefs about capabilities; motivation and goals; memory, social influences; attention and decision processes; behavioural regulation; environmental context and resources; emotion; and nature of the behaviours among others(Atkins et al., 2017). The theoretical domains framework can either be used before the implementation of an intervention / trial or after the programme or trial. This then helps in identifying how to implement an intervention better. (Atkins et al., 2017).

The health belief theory

Perceived susceptibility. This looks at the subjective perception of the risk the patient is at from a state or condition.

Perceived severity. This refers to subjective evaluation of the seriousness of the consequences that could be related to the state or condition.

Perceived threat. The product/sum of severity and susceptibility.

Perceived benefits. This refers to the subjectively understood positive benefits of taking an initiative to mitigate the threat.

Perceived barriers. These are the perceived negatively valued aspects of acting, or counteracting anticipated barriers to taking it.

Self-efficacy. This refers to belief in one's ability to perform or carry out a given behaviour.

Expectations. They are outcomes of perceived benefits, barriers and self-efficacy.

Cues to action. Reminders or prompts to take actions consistent with an intention, ranging from advertising to personal communications from health professionals, family members and/or peers.

Demographic and socio-economic variables. These include age and ethnicity

(University of Twente, 2017).

The theoretical domains framework and the health belief theory will be used in exploring the perceptions of the health workers on screening for depression.

1.07 Conceptual framework of screening for depression strategy.

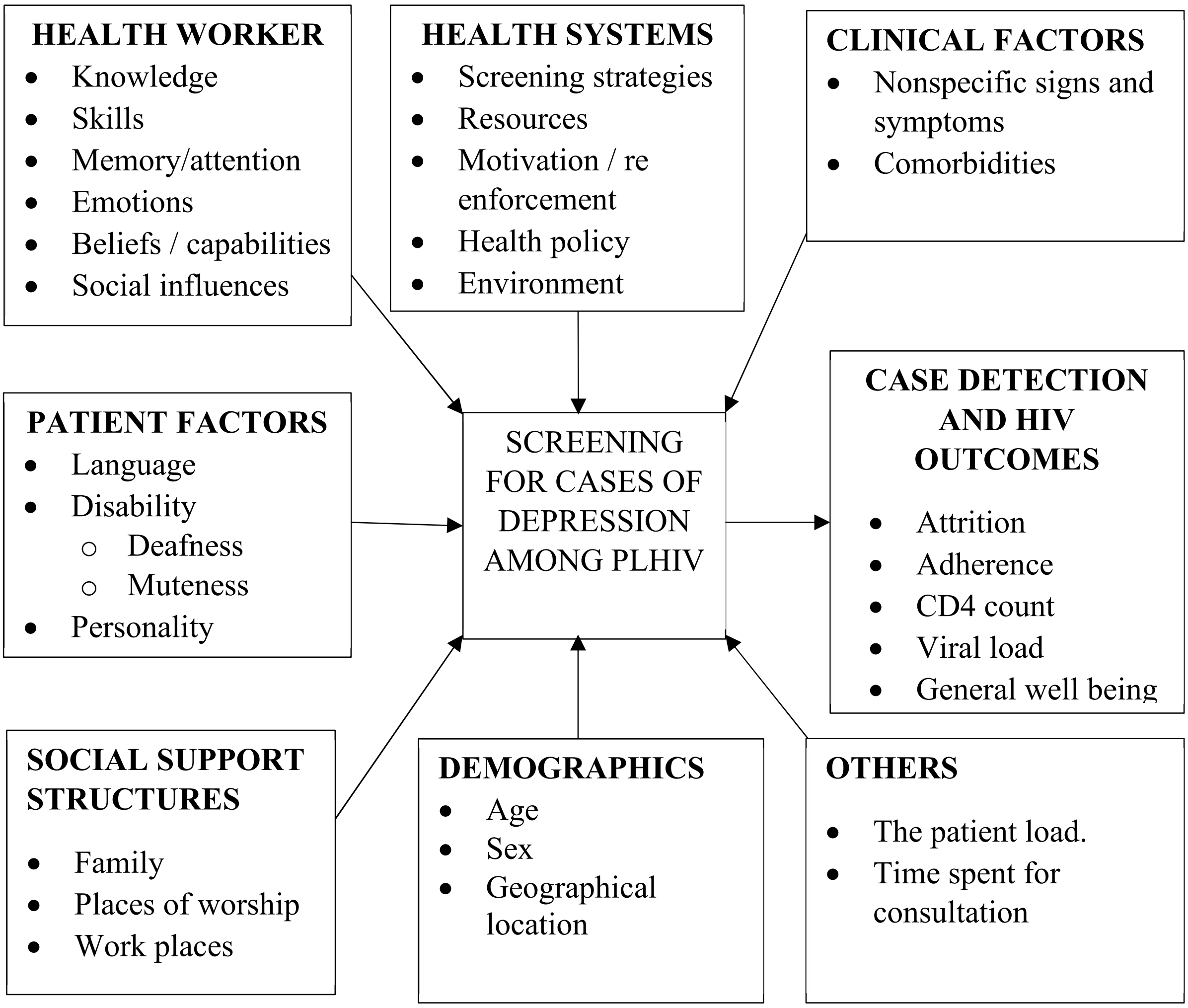

Figure 1: Showing conceptual framework of screening strategy for depression.

Scope of the study

There are several factors that affect screening for depression as shown above (figure 1). All the domains listed above will be studied apart from social support structures and long-term outcomes of management of depression, and the effects of depression management on HIV screening. The outcome variables of interest are cases of depression detected among PLHIV (primary) and the missed cases of depression by the two strategies (secondary).

For the qualitative study the outcome variable is perceptions of stake holders on the screening strategies.

CHAPTER TWO

2.00 Literature review

2.01 Depression

Depression is a common disorder characterised by low mood, loss of interest and enjoyment and reduced energy leading to diminished activity and in severe forms, difficult day-to-day functioning.(MOH Uganda, 2016b). Symptoms of clinical depression are in two categories: affective and somatic. Affective symptoms include depressed mood, loss of interest in normally pleasurable activities, feelings of guilt or worthlessness, hopelessness or suicidal ideation. Somatic symptoms include loss of weight or appetite, sleep disturbances, agitation / retardation, fatigue and loss of concentration(American Psychiatric Association, 2012; MOH Uganda, 2016b). The clinical features of depression are known to be the same in both HIV positive and negative individuals. However, the insomnia and loss of appetite is known to be more frequent among HIV positive as compared to the HIV negative(Melis, Usach, Gandía, & Peris, 2016). With time, these effects of depression affect general health.

Depression is associated with a number of factors such as social isolation, age, poverty, HIV infection, health worsening, some ARVs, lower CD4 count, and major changes in life such as child birth, to mention but a few(Abas, 2014). Depression is known to be linked to physical symptoms given that both physical symptoms and depression are influenced by the neurotransmitters serotonin and norepinephrine. Dysregulation of these transmitters therefore is linked to both depression and pain. It is because of the link between depression and physical symptoms that depression is often times missed by primary health care workers(Trivedi, 2004). Ever since the advent of ART, the life expectancy of PLHIV has improved with more HIV infected people living longer in a better state of health. Initiation to ART has been associated with reduced depression. However, some ARVs such as stavudine and efavirenz have been

found to be associated with depressive symptoms and could be triggering depression among PLHIV(American Psychiatric Association, 2012).

2.02 Screening for depression

Screening for depression is done through use of screening tools, history taking and examinations. A number of tools are used to screen for depression and this variability in the tools have partly contributed to the high variability in the prevalence of depression in various settings(Charlotte et al., 2017). Some of the tools include; the Mini International Neuropsychiatric Interview (M.I.N.I), the 2 and 9 item Patient health questionnaires (PHQ2 and PHQ9).

The M.I.N.I is a short diagnostic structured interview developed to explore 17 disorders according to Diagnostic and Statistical Manual (DSM)-III diagnostic criteria. It is fully structured to allow administration by non-specialized interviewers (Sheehan DV & Lecrubier Y, 1992).

The PHQ-9 is a 9-item self-rating scale evaluating the key symptoms of depression during the past two weeks. The total score ranges from 0 to 27 with five categories of severity: minimal (0–4), mild (5–9), moderate (10–14), moderately severe (15–19) and severe (20–27), which guide in grading depression (MOH Uganda, 2016). The tool has a sensitivity of 91.6%, and specificity of 81.2% (Akena, Joska, Obuku, & Stein, 2013)

The PHQ-2 tool is a shorter version of the PHQ-9. It contains the first two questions found in the PHQ-9. The PHQ-2 score ranges between 0–6 and those with a score equal or greater than 3 are further evaluated using the PHQ-9. The PHQ-2 has a sensitivity and specificity of 82.9% and 90% respectively at a score of 3 (Kroenke, 2003). The PHQs however is mainly recommended for people aged 12 years of age and above(University of Washington, 2017)

Other tools include the Centre for Epidemiologic Studies Depression Scale (CES-D); the Hopkins Symptom Checklist (HSCL), among others.

Though a number of tools are used in screening for depression, the PHQ, was recommended for use in PLHIV populations by a number of studies as a result of its performance in screening for depression among PLHIV. The PHQ performed with an accuracy of 96%, sensitivity of 91.6% and a specificity of 81.2%(Akena et al., 2013). The PHQ has a number of advantages which include; it is shorter than other depression rating scales, can be administered by a clinician, by telephone, or self-administered, facilitates diagnosis of major depression, provides assessment of symptom severity, is well validated and documented in a variety of populations and can be used in adolescents as young as 12 years of age(University of Washington, 2017)

2.03 Approaches to screening

Universal approach versus Sub population approach to screening

Population based screening focuses on the screening the entire population for example the entire population seeking care in the health facility. It is known to yield a number of cases of depression. However, universal screening has been found to have short comings when it comes to applicability in resource limited settings of which there may not be a possibility of adequate follow up and treatment, increased work burden,(Community Care of North Carolina, 2016) and the possibility of the screening leading to a number of false positives.

Sub-population screening on the other hand focuses on a section of the population for example those at high risk of depression such as; PLHIV, the pregnant, postnatal women, and the elderly. Sub-population screening has been argued to be time saving living more time for other important activities within the population, it is also thought to lead to less false positives(Community Care of North Carolina, 2016).

Organised versus Opportunistic screening

Organised screening programmes ensure that every participant is offered the same services including information and support. In most cases, large numbers of people are mobilised to take part in organised screening programmes(New Zealand Government, 2014). This however is not recommended by some governments as there is a possibility of high false positives if applied to the general population.

Opportunistic screening on the other hand happens when a patient or client asks their doctor, clinician or the attending health professional for a check or test, or a check or test is initiated by a doctor or health professional when he finds it appropriate. However, unlike organised screening where the program is monitored or supervised, the alternate opportunistic screening may not be supervised or monitored (New Zealand Government, 2014)leaving challenges with surveillance.

2.04 Studies done on screening for depression strategies / approaches.

There is a limited number of trials done to identify approaches that could increase the detection of depression among high risk populations including PLHIV.

In a developed country one study focused on comparing routine screening with usual practice (clinical signs and symptoms) in the high risk groups (Romera et al., 2013) while another focused on increasing detection through the use of a depression sensitive electronic booking system among pregnant women(Carroll, et al, 2013).

The pragmatic cluster randomised trial by Romera et al (in Spain) randomised the health workers to the control and intervention group. The health workers delivering the intervention screened the patients thought to be at high risk for depression. "High risk" was defined as fulfilling at least one of the following: history of depression, somatic symptoms without any cause, psychological comorbidities or drug abuse, or chronic pain. These patients were

evaluated using two questions: “Over the past two weeks, have you felt down, depressed, or hopeless?” and “Over the past two weeks, have you felt little interest or pleasure in doing things?”. The patients were further evaluated using a diagnostic interview.
The control was subjected to usual clinical practice procedures. There were however un able to detect a difference in cases of depression detected. This was attributed to the failure of the intervention arm to adhere to the screening strategies.

A study done in a developing country in Africa (Kenya) focused on the training the health workers to equip them with skills and knowledge on depression. The patients from the intervention clinics were trained in diagnosis of mental health conditions and allowed to offer mental health services on a daily basis. This study however found no difference in cases of depression of mental health (including depression) detected between the control and intervention (Jenkins et al., 2013). This study didn’t however describe the strategy used.

A prospective observational, quasi-experimental study done by Schumacher and colleagues in a cohort of HIV patients, routinely screened and followed patients for 4 to 6 months. The results obtained were compared against the baseline results. The study demonstrated routine screening for depression can not only identify patients with depression but also patients at risk of other psychiatric conditions. This study was done in the United States of America in a HIV clinic cohort. This study was however prone to confounding bias and the study focused on only one clinic.

There is however hardly any trial comparing selective to routine screening strategy. However, a number of studies done have focussed on the effect of notifying the health workers about the patient’s depression risk status. In these studies, Patients with depression identified in clinics used to be randomised to either the intervention or control. The intervention arm was notified about the patients’ depression risk status while the while the control was not. A meta-analysis

on the effects of notifying health workers on the patients' depression risk status showed that the case detection improved by a range of 10% to 20% (Pignone et al., 2002). However, these studies were done in developed countries among general patients not specifically the PLHIV. These studies however focussed on notification and not screening procedures. However, it is important to note that the study brings out the fact that frequent notification increases diagnosis and treat of depression. A strategy that could therefore bring about more impressions of depression could be of benefit to the people of risk.

2.05 Perceptions of depression screening

There is scarcity of literature on the perceptions of primary health workers on the screening for depression among PLHIV.

In the United States of America (USA), preventive task force recommends screening for depression in the general population of adults, including pregnant and postpartum females. The task force however recommended screening to be implemented with adequate systems in place to ensure accurate diagnosis, effective treatment, and appropriate follow-up. However, some countries such the United Kingdom do not find it appropriate to screen for depression in the general population citing high false positives as the reason (USPTF, 2017).

In Africa, a study in Zimbabwe found the following issues critical and could interfere with the implementation of mental health care. These include: lack of training in mental health, low clinical staff levels, unavailability of psychiatric drugs, unavailability of time for counselling, and poor and unreliable referral systems for people suffering with depression(Chibanda et al., 2016). The shortcoming is however the absence on the view points in the domains of self-efficacy (beliefs about capabilities), beliefs about consequences of screening (anticipated outcomes/ attitudes), memory / attention / decision process, social influences, emotions, behavioural regulation, and roles and identity of the health workers.

CHAPTER THREE

3.00 METHODS

3.01 Study design

A mixed methods study design with quantitative and qualitative data collection methods.

Objective one

A randomised controlled trial design will be used to determine whether there is a difference in cases of depression detected when using routine or selective screening strategies.

Objective two

A qualitative study employing key informant interviews among HIV health care providers.

3.02 The study setting.

The study setting is Princess Diana Memorial Health Centre IV is a government health facility found in Soroti district found in the north-east of Uganda. Soroti district has a population of 296,833. The prevalence of HIV in north-east Uganda is 3.7%. Princess Diana Memorial Health Centre IV is located in a peri-urban setting within northern division along Soroti-Moroto road. Northern division has a population of 19,382 (UBOS, 2014). Princess Diana Memorial Health Centre IV has a catchment population of approximately 11,500 people. The health unit has approximately 470 HIV positive patients with two clinic days per week with an attendance ranging from 20 to 35 patients per clinic day. The health centre is staffed with two psychiatric nurses serving the mental clinics in addition to the other non-psychiatric staff.

Methods for objective one

In order to determine whether there is a difference in cases of depression detected when using routine or selective screening strategies among PLHIV, a randomised controlled trial will be used.

3.03 Population

Target population

All PLHIV aged 18 years and above attending the HIV clinics in Soroti district.

Accessible population

PLHIV aged 18 years and above attending the HIV clinics in Princess Diana Memorial health centre IV site during the study period (January 2018 to March 2018).

Study population

3.04 Eligibility criteria

Inclusion criteria

All PLHIV aged 18 years and above attending the HIV clinics in the study site during the study period and who consent to participate in the study.

Exclusion criteria

PLHIV who are too ill to withstand the study procedures will be excluded from the study.

3.05 Sample size estimate

The following formula will be used; $n = [Z_{\alpha} \sqrt{(2p_1(1\text{-}p_1))} - Z_{\beta} \sqrt{(p_1(1\text{-}p_1) + p_2(1\text{-}p_2))}]^2 / (p_1\text{-}p_2)^2$, (Dawson & G Trapp, 2004) where 'n' is sample size per group 'Z_{α}' is the critical value of the Normal distribution at 'α', 'Z_{β}' is the critical value of the Normal distribution at 'β' and 'p_1' and 'p_2' are the expected sample proportions of the two groups.

Assuming 95% of the cases of depression are missed in primary care practice by selective screening (Jenkins et al., 2013) and we would like to notice a 10% reduction in the cases missed (Pignone et al., 2002).

$(P_1 - P_2)/ P_1 = 0.1$

$P_2 = P_1 – (P_1 * 0.1)$

$P_2 = 0.95 – (0.95*0.1) = 0.855.$

$n = [Z_\alpha \sqrt{}(2p_1(1-p_1)) - Z_\beta \sqrt{}(p_1(1-p_1) + p_2(1-p_2))]^2 / (p_1-p_2)^2$

$n = [1.96 \sqrt{} (2*0.95(1-0.95)) – (-1.282) \sqrt{} (0.95(1-0.95) + 0.855(1-0.855))]^2 / (0.95-0.855)^2$

n=142.74 ~ 143 PLHIV per group and 286 in two groups

Catering for non-response at 20% per group,

0.20*143 = 28.6 ~ 29.

Therefore, a total sample size of 344 patients will be enrolled for the study with 172 PLHIV per group.

3.06 Sampling procedure

The patients will be enrolled consecutively as they fulfil the eligibility criteria since the number of patients per clinic day (20 to 35) can be evaluated by the study team of one principal investigator and four clinical research assistants.

3.07 Randomisation

Randomisation of the patients to the intervention and control will be performed at individual level using random block sizes of 4, 6, 8, 10, and 12. The randomisation code will be generated by an independent statistician using a computer software called Random Allocation Software version 1.0.0

Concealment of the randomisation code

The codes will be concealed using the sequentially numbered opaque sealed envelopes and kept with the Nurse In-charge who will pick an envelope for each patient that is enrolled in the study using the eligibility criteria.

Blinding

The health workers who will be administering the intervention and the control will not be blinded given that it is not feasible to blind them. However, the research assistant (psychiatric nurse) administering the M.I.N.I., and the patients will be blinded. The research assistant administering the M.I.N.I will not be told which study groups the patients were allocated. The patients too will not be told which arm they have been allocated to.

In addition, the research assistant administering the M.I.N.I will not have access to the presumptive diagnoses from the research assistants administering the PHQ 2 and 9.

3.08 Study variables

The intervention

The intervention will be comprised of routine screening. In this strategy PLHIV who consent will be subjected to the PHQ-2 and if the patient has a value greater than or equal to 3 in the PHQ-2 the patient will be subjected to PHQ-9. After wards all patients will be subjected to the M.I.N.I to confirm cases of major depressive disorder (MDD), and to identify the missed cases of MDD. The M.I.N.I will be used to identify the actual number of cases of MDD in the group.

The control

The control arm will provide the standard of care which is selective screening. A patient who consents to the study will be subjected to the PHQ2 and later (if PHQ2 $\geq$3) PHQ 9 when the clinician finds it appropriate especially at the crisis points in life in accordance with the ministry of health 2016 guidelines for prevention and treatment of HIV in Uganda. The clinician will require to indicate the reason for screening before screening for depression.

After wards all patients will be subjected to the M.I.N.I to confirm cases of major depressive disorder (MDD), and to identify the missed cases of MDD. The M.I.N.I will be used to identify the actual number of cases of MDD in the group.

The crisis points in life include: newly diagnosed with HIV or at disclosure of HIV status; occurrence of any physical illness, recognition of new symptoms / progression of disease or hospitalisation or diagnosis of AIDs; introduction to medication; death of a significant other; necessity of making end of life; and permanency planning decision, major life changes like child birth, pregnancy, loss of a job, and end of a relationship.

Outcome variable

The outcome variable of interest is the percentage of missed cases of depression.

The potential confounders

Basing on literature, the following potential confounders will be considered; age (Payne, 2017) , marital status (Bulloch, Williams, Lavorato, & Patten, 2017), sex (Karger, 2014), weight (Tracy, 2017), viral load (J Peters, 2014).

3.09 Data collection

Data collection procedure

The study team will comprise of the principal investigator, two psychiatric nurses, two clinical officers, one social worker and two data entrants

Data will be collected in three consultation rooms. Patients in the intervention arm will be evaluated by a clinician stationed in a consultation room dedicated to the intervention arm. Patients in the control arm will be evaluated by a clinician in a separate room dedicated to the control arm. Finally, all patients will be evaluated by a psychiatric nurse using the MINI in the third consultation room.

Measurement of variables

Case detection: These will be measured using the PHQ-2 and the PHQ-9 questionnaires and graded using the guide for diagnosis and management of depression adopted from the Ministry of Health guidelines 2016.

The cases missed by the strategies will be detected using the M.I.N.I. It will therefore be used to determine the denominator (cases of depression present) in both groups.

The PHQ-2 tool is a two-item instrument that inquiries about the frequency of depressed mood and anhedonia over the past two weeks. The purpose of the tool is to screen for depression in a first step approach. The PHQ-2 score ranges between 0–6 and those with a score equal or greater than 3 are further evaluated using the PHQ-9.

The PHQ-9 is a 9-item depression screening instrument that determines the presence and frequency of the 9 core depressive symptoms identified in the DSM-IV over the previous 2 weeks. This tool has been used in sub-Saharan Africa for a number of studies(Akena et al., 2013). Scores range from 0–27, with a score of 10 or higher usually used to indicate the presence of a depressive disorder that would benefit from treatment. The PHQ-9 was developed to be self-administered, however interviewer-administration and telephone administration (Pence et al., 2012) has yielded similar results.

MINI—The Mini International Neuropsychiatric Interview (MINI) is a brief, structured diagnostic interview for major psychiatric disorders. The MINI will serve as the reference standard in this study which will be used to determine the cases in the denominator. The MINI modules for major depressive disorder (MDD) will be used for this study.

3.10 Data management

Emphasis will be made on filling questionnaires correctly. The filled questionnaires will be kept securely under lock and key. This will be entered using Epidata by two independent data entrants. The data will be exported to Stata 13 software. The data will be backed up in google drive and an external drive.

3.11 Data analysis

Skewed data will be summarised using median and quartiles while mean and standard deviation will be used to summarise non-skewed data.

The trial data will be analysed on an intention to treat analysis basis.

Two-Sample Z-Test for proportions will be used to test the single difference between proportions of cases detected in the two study arms (Dawson & G Trapp, 2004) if the data meets the conditions for the statistic otherwise chi square statistics or Fisher's exact test will be used to answer the objective. Missed cases proportions in a group will be calculated as follows,

$$z = \frac{\hat{p}_1 - \hat{p}_2}{SE} = \frac{\hat{p}_1 - \hat{p}_2}{\sqrt{\hat{p}(1-\hat{p})\left(\frac{1}{n_1}+\frac{1}{n_2}\right)}}$$

$\hat{P} = (\hat{P}_1 n_1 + \hat{P}_2 n_2) / (n_1 + n_2)$

Where;

$\hat{P}_1$ is proportion in group one

$\hat{P}_2$ is proportion in group two

$\hat{P}$ is the pooled proportion of $\hat{p}_1$ and $\hat{p}_2$

n_1 is the sample size of group one

n_2 is the sample size of group two.

The estimates' level of significance will be ascertained using the 95% confidence interval and P-value. The estimate will be considered significant if an estimate's confidence interval does not contain the null value and if the p value is less than 0.05.

Methods for objective two

In order to describe the perceptions of stakeholders on the screening strategies for depression among PLHIV, qualitative data collection methods namely key informants' interviews will be conducted.

3.12 Participants

The 5 health care workers who participated in the study will be requested to participate in the qualitative study. The PLHIV will be interviewed until a point of saturation is reached.

3.13 Sampling procedure

Purposive sampling will be used in the study.

Only the health workers who are linked to the HIV service provision will be interviewed.

3.14 Study variables

Outcome variable:

The outcome variable of the qualitative study is perception of stake holders about the screening strategies.

Predictor variables

<u>Socio-demographics:</u> age, sex, cadre, education level.

<u>The screening for depression experience</u>: convenience, and time to results.

3.15 Data collection

Perceptions

Perceptions will be considered to be opinions, thoughts, views, beliefs or feelings about the screening strategies of the stake holders.

Interview guides for will be used by a social worker to interview the perceptions of health workers on the screening strategies. The in-depth interviews will be voice recode using a recorder or a computer application to ensure that the details of the interview are not missed.

3.16 Data management

Emphasis will be placed on completely filling the questionnaires and ensuring that in-depth interviews are voice recorded. The filled data collection materials will be kept under lock and key. Data will be entered in the computer using Epi data and latter exported to excel for

cleaning. This data including recoded interviews will be backed up in the google drive and external drive for safe storage.

3.17 Data analysis

The written information from the interviews will be enriched by the audio-taped key informant interviews which will be transcribed verbatim before editing to remove any identifiers. Transcripts will be read thoroughly and over multiple times and coded independently by two study team members trained and with experienced in qualitative data analysis. Information from the in-depth interviews will then be coded manually by two independent analysts trained in qualitative data analysis using a thematic approach by identifying, analysing and reporting patterns (themes) within data to bring out the detail. The purpose of bringing out the detail is to illuminate the specific, to identify phenomena through which the clinicians perceived the screening situations. The information from the qualitative data will be triangulated with the quantitative data.

3.18 Quality control

The following will be considered in quality control

- Prior training of all the team members on the data collection will be done.
- Patient questionnaires will be checked for completeness and error daily for rectification before breaking off and storage.
- Double data entry. Discrepancies will be rectified by consulting the original questionnaires.
- Participants (patients) will have unique numbers for identification and avoiding data contamination
- Participants will be led by a research assistant to the allocated room to ensure participants remain in the respective arms.

- The clinicians administering the standard of care (control) will indicate the reason for screening for depression to avoid screening for in eligible patients. This will be indicted on the provided checklist.
- Health workers (Clinical research assistants) will not be allowed to switch roles.
- Questionnaires will be translated to Ateso and back translated to English to ensure consistency in the message
- Questionnaires will be pretested before use in the field.

3.19 Ethical Considerations

- Permission will be sought from the Clinical Epidemiology Unit (CEU) before submitting it to School of Medicine Research and Ethics Committee (SoMREC) and Uganda National Council of Science and Technology (UNCST) for ethical approval.
- Administrative permission from Soroti District Local Government (SDLG) and the Incharge Princess Diana Memorial Health Centre IV will be sought for before commencing with the study.
- In addition, informed consent will be obtained from all patients before enrolment in the study.
- Work will be carried out with the specialised psychiatric nurses in addition to the HIV care providers.
- Patients found to have depression will be managed according to the standard of care (following the Ministry of Health guidelines).

3.20 Limitations of the study

- Assessing for the contribution of annual screening in detecting depression will not be possible.

3.21 Dissemination plans

Results from this study will be presented to the Clinical Epidemiology Unit. The dissertation will be submitted to; Clinical Epidemiology Unit, Directorate of Research and Graduate Training of Makerere University, and Sir Albert Cook Library-College of Health Sciences, and

the Soroti District Local Government, Princess Diana Health Centre IV, and the Ministry of Health. Additionally, study findings will be published in a peer reviewed journal and presented in conferences.

References

Abas, M. A. (2014). Depression and HIV Time to Act. Retrieved from http://www.academia.edu/9746113/Depression_and_HIV_Time_to_Act

Abebaw, F. (2017). Recognition of depression by primary care clinicians in rural Ethiopia. Retrieved November 10, 2017, from https://www.ncbi.nlm.nih.gov/pmc/articles/PMC5399858/

Akena, D. (2013). A Comparison of Five Brief Screening Tools for HIV-Associated Neurocognitive Disorders in the USA and South Africa | SpringerLink. Retrieved November 10, 2017, from https://link.springer.com/article/10.1007/s10461-016-1316-y

Akena, D., Joska, J., Obuku, E. A., & Stein, D. J. (2013). Sensitivity and specificity of clinician administered screening instruments in detecting depression among HIV-positive individuals in Uganda. *AIDS Care*, *25*(10), 1245–1252. https://doi.org/10.1080/09540121.2013.764385

American Psychiatric Association. (2012). FactSheet-Depression-2012.pdf.

Atkins, L., Francis, J., Islam, R., O'Connor, D., Patey, A., Ivers, N., … Michie, S. (2017). A guide to using the Theoretical Domains Framework of behaviour change to investigate implementation problems. *Implementation Science*, *12*, 77. https://doi.org/10.1186/s13012-017-0605-9

Benton, T. D. (2008). Depression and HIV/AIDS. *Current Psychiatry Reports*, *10*(3), 280. https://doi.org/10.1007/s11920-008-0045-y

Bulloch, A. G. M., Williams, J. V. A., Lavorato, D. H., & Patten, S. B. (2017). The depression and marital status relationship is modified by both age and gender. *Journal of Affective Disorders*, *223*(Supplement C), 65–68. https://doi.org/10.1016/j.jad.2017.06.007

Caballero, L., Aragonès, E., García-Campayo, J., Rodríguez-Artalejo, F., Ayuso-Mateos, J. L., Polavieja, P., … Gilaberte, I. (2008). Prevalence, characteristics, and attribution of somatic symptoms in Spanish patients with major depressive disorder seeking primary health care. *Psychosomatics*, *49*(6), 520–529. https://doi.org/10.1176/appi.psy.49.6.520

Carroll, A. E., Biondich, P., Anand, V., Dugan, T. M., & Downs, S. M. (2013). A randomized controlled trial of screening for maternal depression with a clinical decision support system. *Journal of the American Medical Informatics Association*, *20*(2), 311–316. https://doi.org/10.1136/amiajnl-2011-000682

Charlotte, B., Dabis, F., & de Rekeneire, N. (2017). Prevalence and factors associated with depression in people living with HIV in sub-Saharan Africa: A systematic review and meta-analysis. *PLOS ONE*, *12*, e0181960. https://doi.org/10.1371/journal.pone.0181960

Chibanda, D., Verhey, R., Munetsi, E., Rusakaniko, S., Cowan, F., & Lund, C. (2016). Scaling up interventions for depression in sub-Saharan Africa: lessons from Zimbabwe. *Global Mental Health*, *3*. https://doi.org/10.1017/gmh.2016.8

Community Care of North Carolina. (2016). Implementation-Guide-for-Depression-Screening-and-Treatment.pdf. Retrieved November 10, 2017, from http://consortiumforis.org/wp-content/uploads/2016/10/Implementation-Guide-for-Depression-Screening-and-Treatment.pdf

Curran, J. A., Brehaut, J., Patey, A. M., Osmond, M., Stiell, I., & Grimshaw, J. M. (2013). Understanding the Canadian adult CT head rule trial: use of the theoretical domains framework for process evaluation. *Implementation Science : IS*, *8*, 25. https://doi.org/10.1186/1748-5908-8-25

Dawson, B., & G Trapp, R. (2004). *Basic & Clinical Biostatistics, 4e | AccessMedicine | McGraw-Hill Medical* (fourth edition). McGraw-Hill Companies, Inc. Retrieved from https://accessmedicine.mhmedical.com/book.aspx?bookID=356

Fact sheet - AIDS epidemic status. (2016). Retrieved November 9, 2017, from http://www.unaids.org/en/resources/fact-sheet

Gaynes, B. N., Pence, B. W., Atashili, J., O'Donnell, J. K., Njamnshi, A. K., Tabenyang, M. E., … Ndumbe, P. (2015). Changes in HIV Outcomes Following Depression Care in a Resource-Limited Setting: Results from a Pilot Study in Bamenda, Cameroon. *PloS One*, *10*(10), e0140001–e0140001. https://doi.org/10.1371/journal.pone.0140001

Gonzalez, J. S., Batchelder, A. W., Psaros, C., & Safren, S. A. (2011). Depression and HIV/AIDS treatment nonadherence: a review and meta-analysis., Depression and HIV/AIDS Treatment Nonadherence: A Review and Meta-analysis. *Journal of Acquired Immune Deficiency Syndromes (1999), Journal of Acquired Immune Deficiency Syndromes (1999)*, *58*, *58*(2, 2), 181–187. https://doi.org/10.1097/QAI.0b013e31822d490a, 10.1097/QAI.0b013e31822d490a

Goodwin, G. M. (2006). Depression and associated physical diseases and symptoms. *Dialogues in Clinical Neuroscience*, *8*(2), 259–265.

Hartzell, J. D., Janke, I. E., & Weintrob, A. C. (2008). Impact of depression on HIV outcomes in the HAART era. *Journal of Antimicrobial Chemotherapy*, *62*(2), 246–255. https://doi.org/10.1093/jac/dkn193

J Peters. (2014). Depression associated with failure to suppress viral load. Retrieved November 12, 2017, from https://www.healio.com/infectious-disease/hiv-aids/news/online/%7Bdebd4fb2-003a-449f-96ed-e094a5ad2cd1%7D/depression-associated-with-failure-to-suppress-viral-load

Jenkins, R., Othieno, C., Okeyo, S., Kaseje, D., Aruwa, J., Oyugi, H., … Kauye, F. (2013). Short structured general mental health in service training programme in Kenya improves patient health and social outcomes but not detection of mental health problems - a pragmatic cluster randomised controlled trial. *International Journal of Mental Health Systems*, *7*(1), 25. https://doi.org/10.1186/1752-4458-7-25

Karger, A. (2014). [Gender differences in depression]. *Bundesgesundheitsblatt, Gesundheitsforschung, Gesundheitsschutz*, *57*(9), 1092–1098. https://doi.org/10.1007/s00103-014-2019-z

Korstanje, E. (2016). When Mental Illness Is Mistaken for Demonic Possession - Broadly. Retrieved November 13, 2017, from https://broadly.vice.com/en_us/article/nejv8w/when-mental-illness-is-mistaken-for-demonic-possession

Kroenke, K. (2003). The Patient Health Questionnaire-2: validity of a two-item depression screener. - PubMed - NCBI. Retrieved November 10, 2017, from https://www.ncbi.nlm.nih.gov/pubmed/14583691

Melis, V., Usach, I., Gandía, P., & Peris, J.-E. (2016). Inhibition of Efavirenz Metabolism by Sertraline and Nortriptyline and Their Effect on Efavirenz Plasma Concentrations. *Antimicrobial Agents and Chemotherapy*, *60*(2), 1022–1028. https://doi.org/10.1128/AAC.02129-15

MOH Uganda. (2016a). CONSOLIDATED GUIDELINES FOR PREVENTION AND TREATMENT OF HIV IN UGANDA.PDF. MOH.

MOH Uganda. (2016b). Uganda Clinical Guidelines 2016 | Ministry of Health. Retrieved November 10, 2017, from http://health.go.ug/content/uganda-clinical-guidelines-2016

Nakimuli, M. (2011). Prevalence and factors associated with depressive disorders in an HIV+ rural patient population in southern Uganda - ScienceDirect. Retrieved November 10, 2017, from http://www.sciencedirect.com/science/article/pii/S0165032711004228

Nakku, J., Kinyanda, E., & Hoskins, S. (2013). Prevalence and factors associated with probable HIV dementia in an African population: A cross-sectional study of an HIV/AIDS clinic population. *BMC Psychiatry*, *13*, 126. https://doi.org/10.1186/1471-244X-13-126

New Zealand Government. (2014). Organised and opportunistic screening | National Screening Unit. Retrieved November 10, 2017, from https://www.nsu.govt.nz/about-us-national-screening-unit/what-screening/organised-and-opportunistic-screening

Okeke, E. N., & Wagner, G. J. (2013). AIDS Treatment and Mental Health: Evidence from Uganda. *Social Science & Medicine (1982)*, *92*, 27–34. https://doi.org/10.1016/j.socscimed.2013.05.018

Payne, C. (2017, August 4). Depression and anxiety levels increase with age and vary by gender in aging South African population. Retrieved November 12, 2017, from https://www.hsph.harvard.edu/population-development/2017/08/04/depression-and-anxiety-levels-increase-with-age-and-vary-by-gender-in-aging-south-african-population/

Pence, B. W., Gaynes, B. N., Atashili, J., O'Donnell, J. K., Tayong, G., Kats, D., … Ndumbe, P. M. (2012). Validity of an Interviewer-Administered Patient Health Questionnaire-9 to Screen for Depression in HIV-Infected Patients in Cameroon. *Journal of Affective Disorders*, *143*(1–3), 208–213. https://doi.org/10.1016/j.jad.2012.05.056

Pignone, M. P., Gaynes, B. N., Rushton, J. L., Burchell, C. M., Orleans, C. T., Mulrow, C. D., & Lohr, K. N. (2002). Screening for Depression in Adults: A Summary of the

Evidence for the U.S. Preventive Services Task Force. *Annals of Internal Medicine*, *136*(10), 765. https://doi.org/10.7326/0003-4819-136-10-200205210-00013

Reynolds, C. F., & Patel, V. (2017). Screening for depression: the global mental health context. *World Psychiatry*, *16*(3), 316–317. https://doi.org/10.1002/wps.20459

Romera, I., Montejo, Á. L., Aragonés, E., Arbesú, J. Á., Iglesias-García, C., López, S., … Gilaberte, I. (2013). Systematic depression screening in high-risk patients attending primary care: a pragmatic cluster-randomized trial. *BMC Psychiatry*, *13*, 83. https://doi.org/10.1186/1471-244X-13-83

Sanganyado, E. (2017, July 24). Why African Christians Should Rethink Depression. Retrieved November 15, 2017, from http://faithfullymagazine.com/african-christians-rethink-depression/

Schumacher, J. E., McCullumsmith, C., Mugavero, M. J., Ingle-Pang, P. E., Raper, J. L., Willig, J. H., … Saag, M. S. (2013). Routine Depression Screening in an HIV Clinic Cohort Identifies Patients with Complex Psychiatric Co-morbidities Who Show Significant Response to Treatment. *AIDS and Behavior*, *17*(8), 2781–2791. https://doi.org/10.1007/s10461-012-0342-7

Sheehan DV & Lecrubier Y. (1992, 2006). MINI_v5_002006.pdf.

Tracy, N. (2017). Depression and Weight Gain, Depression and Weight Loss - Symptoms - Depression. Retrieved November 12, 2017, from https://www.healthyplace.com/depression/symptoms/depression-and-weight-gain-depression-and-weight-loss/

Trivedi, M. H. (2004). The link between depression and physical symptoms. *Primary Care Companion to the Journal of Clinical Psychiatry*, *6*(Suppl 1), 12–16.

UBOS, U. (2014). 2014 National Census Main Report.pdf. Retrieved November 10, 2017, from

http://www.ubos.org/onlinefiles/uploads/ubos/NPHC/2014%20National%20Census%20Main%20Report.pdf

Udedi, M. (2014). The prevalence of depression among patients and its detection by primary health care workers at Matawale Health Centre (Zomba). *Malawi Medical Journal: The Journal of Medical Association of Malawi*, *26*(2), 34–37.

UN health agency, U. N. N. S. (2017, February 23). UN News - UN health agency report. Retrieved November 9, 2017, from http://www.un.org/apps/news/story.asp?NewsID=56230#.WgR65fmWbIU

University of Twente. (2017). Health Communication | Health Belief Model. Retrieved November 10, 2017, from https://www.utwente.nl/en/bms/communication-theories/sorted-by-cluster/Health%20Communication/Health_Belief_Model/

University of Washington. (2017). PHQ-9 Depression Scale | University of Washington AIMS Center. Retrieved November 10, 2017, from https://aims.uw.edu/resource-library/phq-9-depression-scale

USPTF. (2017). Screening for Depression in Adults: US Preventive Services Task Force Recommendation Statement. - PubMed - NCBI. Retrieved November 10, 2017, from https://www.ncbi.nlm.nih.gov/pubmed/26813211

WHO. (2017a). WHO Treatment and Prevention guidelines 2016. Retrieved November 9, 2017, from http://apps.who.int/iris/bitstream/10665/255880/1/9789241550055-eng.pdf?ua=1

WHO | (GHO) data. (2017). Retrieved November 9, 2017, from http://www.who.int/gho/en/

WHO, P. (2017b, August 21). Uganda Population-Based HIV Impact Assessment UPHIA 2016–2017 Fact sheet, August 2017 [Text]. Retrieved November 10, 2017, from https://reliefweb.int/report/uganda/uganda-population-based-hiv-impact-assessment-uphia-2016-2017-fact-sheet-august-2017
